# Traditional Gender Role Beliefs and Career Attainment in STEM: A Gendered Story?

**DOI:** 10.3389/fpsyg.2019.01053

**Published:** 2019-05-08

**Authors:** Anna-Lena Dicke, Nayssan Safavian, Jacquelynne S. Eccles

**Affiliations:** School of Education, University of California, Irvine, Irvine, CA, United States

**Keywords:** traditional gender role beliefs, educational attainment, STEM, occupational attainment, gender differences

## Abstract

Gender role beliefs (i.e., beliefs about gender-specific responsibilities) predict one’s educational and occupational aspirations and choices (Eccles et al., 1983; Schoon and Parsons, 2002). Focusing on STEM careers, we aim to examine the extent to which traditional work/family related gender role beliefs (TGRB) in adolescence predict within and across gender differences in subsequent educational and STEM occupational attainment in adulthood. Using longitudinal data from the Michigan Study of Adolescent and Adult Life Transitions (*N* = 744; 58% female), participants’ educational attainment and their occupations were assessed at age 42. Their occupations were then categorized into three categories: traditional STEM-related careers in the physical sciences, mathematics, engineering, and technology (PMET); life sciences (e.g., health sciences, LS); and non-STEM. For females, TGRB at age 16/18 significantly predicted lower educational attainment as well as a lower likelihood to be in PMET-related occupations in comparison to non-STEM occupations – controlling for their own educational attainment. TGRB also predicted a higher likelihood to be in LS-related in comparison to PMET-related occupations. No significant associations were found for males. However, patterns of findings for males were similar to those of females. TGRB also mediated across gender differences in educational and PMET-related occupational attainment. Findings reveal TGRB to be one underlying psychological factor influencing gender disparity in educational and STEM occupational attainment.

## Introduction

There has been an increasing number of students aspiring to careers in STEM (science, technology, engineering, and mathematics) in the last decade. The STEM workforce is also increasingly diversifying with respects to gender as female students outnumber male students in some STEM fields, such as biology, medicine, and chemistry (Beede et al., 2011). However, females are still underrepresented in engineering, computer science, and physical sciences (Chen and Ho, 2012). A multitude of reasons for the gender disparities in STEM participation have been investigated, including gender differences in attitudes and beliefs, such as the valuing of various STEM domains (Eccles et al., 1993; Ceci et al., 2014; Lauermann et al., 2015; Cheryan et al., 2017). One of the relevant underlying beliefs that might be driving gender differences in STEM participation are traditional gender role beliefs. These general beliefs about responsibilities and behaviors deemed appropriate for women and men (Eccles, 1987; Williams and Best, 1990) predict aspirations, choices, and occupational outcomes (Eccles et al., 1990). However, the long-term impact of traditional gender role beliefs on STEM participation is less understood.

In the current study, we address this gap in research by investigating the long-term association of traditional gender-role beliefs in adolescence with subsequent educational and STEM occupational attainment in adulthood for females and males using a longitudinal dataset spanning over 20 years. To explore the complexity of the impact of traditional gender role beliefs on these outcomes, we investigated the impact of traditional gender role beliefs within as well as across genders.

### Understanding Gender Disparity in STEM Fields

To better address the gender disparities across various STEM fields, the mechanisms behind its emergence need to be better understood. Research has shown that gender differences are evident in the valuing of gender-stereotyped domains such as mathematics and physics with males showing a stronger inclination toward typically male-stereotyped domains and vice versa for females (e.g., Eccles et al., 1993). Similarly, the values underlying various career-related choices are often gendered. For example, females tend to value helping others, improving society, and giving back to their communities relative to males and to other career values such as making lots of money (Lyson, 1984; Eccles, 1987; Konrad et al., 2000). This is consistent with their prevalent interest in human services occupations (Lauermann et al., 2015). Males, on the other hand, are more likely than females to value working with tools and machines and making lots of money, as well as to aspire to careers within traditional male dominant STEM domains (Su et al., 2009; Wang and Degol, 2013; Ramaci et al., 2017).

According to the Eccles et al. (1983) Expectancy-Value theoretical framework, social and contextual factors (such as cultural values, gender role belief systems, social beliefs and behaviors, and prior aptitude and experiences) exert influence onto adolescents’ self-beliefs, aspirations, choices, and attainment through their socialization experiences. Thus, gender differences in valuing and subsequent choices are likely results of internalized cultural values and social expectations linked to such belief systems as gender roles (see Eccles et al., 1983, 1990; Eccles, 2015).

### Traditional Gender Role Beliefs and Educational and STEM Occupational Attainment

Amongst important internalized social and cultural values are the general beliefs about responsibilities and behaviors deemed appropriate for women and men (Eccles et al., 1983; Eagly, 1987; Williams and Best, 1990; Corrigall and Konrad, 2007): Individuals holding traditional gender role beliefs support women’s role as the caretaker at home and in the family and men’s role is to provide financial support as the breadwinner of the family. Research has shown that traditional gender role beliefs are more strongly endorsed by men than women (Larsen and Long, 1988; Brewster and Padavic, 2000). These beliefs are linked to greater emphasis being put on men’s and husbands’/fathers’ careers than on women’s and wives/mothers’ careers. Such beliefs are then likely to be reflected in individual women’s and men’s social identities, anticipated future social roles, and short-and long-term goals (Eccles and Bryan, 1994; Eccles et al., 1999). They are also key predictors of their aspirations and both educational and occupational choices (e.g., Schoon and Parsons, 2002; Webb et al., 2002).

Women who endorse traditional gender role beliefs related to family and work roles are more likely to focus on family responsibilities with consequences for the choices they make with regards to educational and occupational aspirations and attainment. For instance, the decrease in traditional work/family related gender role beliefs within society is likely related to increases in educational attainment for females (Buchmann et al., 2008). Female participation in higher education has increased as the prevalence of traditional family related gender role beliefs decreased over time (Brooks and Bolzendahl, 2004; Goldin, 2006). Furthermore, Scott (2004) found a direct link between traditional gender role beliefs and educational attainment: Using data from a National Panel study in Britain, females holding more traditional beliefs about family and work were more likely to show worse performance in their high school exams than females not endorsing traditional beliefs. As expected, given the emphasis of the males’ role as a breadwinner within the traditional gender role belief system, this association was not as pronounced for males.

Past studies have also shown associations of endorsement of traditional work/family related gender role beliefs with employment and earnings for females (Cassidy and Warren, 1996; Christie-Mizell, 2006; Corrigall and Konrad, 2007; Buchmann et al., 2008). For instance, Corrigall and Konrad (2007) found that women with more traditional attitudes in their early twenties worked fewer hours and had lower income than women with more egalitarian views in their late twenties using a large nationwide United States sample. In addition, Christie-Mizell (2006) found that endorsement of traditional gender role beliefs was most strongly associated with a decrease in income for white women compared to white and black men and black women within a large-scale longitudinal United States sample.

Although traditional gender role beliefs have become less prevalent over time (Bolzendahl and Myers, 2004; Brooks and Bolzendahl, 2004; Raley et al., 2006), these core beliefs about the roles of women and men in society might help explain still existing differences in STEM occupational choices across gender. According to the Expectancy-Value theoretical framework (Eccles et al., 1983), links between gender role belief systems operate through the association of gender role beliefs with both individuals’ gendered expectations for success in and the relative attainment values of various gender typed occupations. Thus, traditional gender role beliefs likely drive across gender differences in STEM-related occupational attainment. With males typically holding more traditional gender role beliefs, they are more likely to seek out high status jobs and thus, pursue STEM-related careers than females, in particular in the traditional STEM fields.

However, the impact of traditional gender role beliefs is likely to be even more complex and might be able to also explain within gender variation in STEM occupational choices. Females are overrepresented in the medical, social, and life sciences, which concern caring and helping others – a value typically endorsed by women with more traditional work/family related gender role beliefs. Females’ interest in these specific STEM fields may be due to the values they attach to these specific fields and the extent to which they identify with these values more than other science disciplines. Thus, a stronger endorsement of traditional work/family related gender role beliefs might be perceived to be in accordance with the pursuit of STEM occupations in the life and medical sciences. In contrast, the more traditional STEM fields, such as physics and engineering, are perceived as male-dominated, isolated, and incompatible with the goals of helping others (Eccles et al., 1999; Cheryan et al., 2015). In other words, traditional gender role beliefs should lead those females who go into STEM to be more likely to go into careers in the medical and life sciences than into more traditional STEM fields. The extent to which traditional gender role beliefs can help explain the unequal distribution of females and males in various STEM fields has not been investigated.

Previous research, however, has shown that females with more traditional work/family related gender role beliefs are less likely than males to persist in STEM occupational aspirations than non-STEM occupational aspirations. In a study using earlier waves of the MSALT dataset used in the present study, Frome et al. (1996) found that traditional work/family gender role beliefs held at age 20 were significantly associated with changes in STEM-related occupational aspirations for females. More specifically, they found that females with more traditional gender role beliefs were more likely to change from an occupational aspiration in math, engineering or physical science in 12th grade to an occupational aspiration outside of these fields at age 20. These links were not found for males. Given the wide variation of STEM and non-STEM careers that fit with male gender roles, the association of traditional work/family related gender role beliefs with within gender variation of occupational choices for males is likely to be less pronounced. Frome et al. (2006) found that the impact of traditional work/family related gender role beliefs persisted for females in a follow-up study of a subsample of females that aspired to male-dominated occupational fields in 12th grade. A higher desire for a family flexible job reported in 12th grade was associated with a change of aspirations away from male-dominated occupational fields by age 25.

In sum, gendered beliefs about suitable social roles inform both the pathways and opportunities that are perceived as accessible or socially desirable, as well as the related educational and occupational choices that young people make along the way toward professional attainment. However, despite some exceptions (Frome et al., 1996; Corrigall and Konrad, 2007), more longitudinal studies investigating the long-term associations of traditional gender role beliefs are needed. In addition, there is a lack of studies investigating the associations of traditional gender role beliefs with gendered patterns of STEM-related occupational attainment using a differentiated conceptualization of traditional STEM fields and medical and life sciences.

### Current Study

In the current study, we address these gaps in existing research by examining a developmental model spanning over 20 years investigating the association of traditional work/family related gender role beliefs in adolescence with educational and STEM-related occupational attainment in adulthood. Furthermore, to accurately capture the representation of males and females in various STEM fields, we created and used a classification of STEM occupations that differentiates the classic STEM disciplines (i.e., physical sciences, engineering, mathematics, and technology, PMET) from the life and medical sciences (LS). In addition, to account for the impact of participants’ socio-demographic family background, we included mother’s educational background as a predictor of participant’s educational and occupational attainment. Using a longitudinal dataset and building on work from Frome et al. (1996), we asked the following research questions:

RQ1: To what extent do traditional gender-role beliefs held in adolescence (age 16/18) predict subsequent educational and STEM occupational attainment in adulthood (age 42) for females and males?

Taking into account the within gendered pattern of occupational choices found in previous work by Frome et al. (1996), we first investigated the associations of traditional gender role beliefs and subsequent educational and STEM occupational attainment separately for male and female adolescents. Based on previous research (e.g., Scott, 2004), we hypothesized that stronger endorsements of traditional gender role beliefs during adolescence would be associated with lower levels of education in adulthood (as measured by years of formal education) amongst females, but not males. We hypothesized that stronger endorsement of traditional gender role beliefs in adolescence would be associated with a reduced likelihood of occupational attainment within male-typed STEM domains (i.e., PMET) compared to non-STEM occupations amongst females, but not males. We also hypothesized that stronger endorsement of traditional gender role beliefs in adolescence would increase the likelihood to be in less male-typed STEM domains (i.e., LS) compared to non-STEM careers. Lastly, we hypothesized that traditional gender role beliefs in adolescence would decrease the likelihood of occupational attainment in less male-typed STEM domains (i.e., LS) relative to male-typed STEM domains (i.e., PMET).

RQ2: Are gender differences in educational and STEM occupational attainment in adulthood (age 42) mediated by traditional gender role beliefs in adolescence (age 16/18)?

We hypothesized that across gender differences in educational and STEM occupational attainment will be mediated by traditional gender role beliefs. Given previous research (Brewster and Padavic, 2000), we hypothesized that males will hold more traditional gender role beliefs than females. Thus, we hypothesized that gender differences in the endorsement of traditional gender role beliefs by males than females will explain differences in rates of educational attainment and STEM-related occupational attainment between males and females. More specifically, we hypothesized that stronger endorsement of traditional gender role beliefs by males will explain a higher rate of attainment of PMET-related compared to non-STEM occupations. In contrast, we hypothesized that a higher rate of attainment of LS-related compared to non-STEM occupations of females will be explained by males’ higher levels of traditional gender role beliefs. The same holds true for the comparison of LS-related and non-STEM related careers.

## Materials and Methods

### Sample

The current study used data from the large scale longitudinal Michigan Study of Adolescent and Adult Life Transitions (MSALT) that followed 2,474 participants over a time span of 30 years from the end of elementary school at age 11 into adulthood at age 42. Participants were from largely middle-income communities located within a large industrial Midwestern city in Michigan, United States and largely from European American descent (91%). We used parent reported data from Wave 1 (participants in grade 6/age 12) and participant self-reported data from Waves 5 and 6 (grade 10/age 16 and grade 12/age 18), and Wave 10 (age 42). In Wave 10, data was collected through surveys via mail, via phone interviews and via web search using social media profiles (i.e., LinkedIn, Facebook). For participants located through web search, educational and occupational attainment was assessed using the information presented in online profiles. All Wave 10 participants with valid data for occupational attainment were included in the current study (*n* = 744; 58% female; 93% European American). This subsample constituted 89% of the overall Wave 10 sample and 30% of the original sample. Attrition analyses using the original sample of 2,474 participants showed that Wave 10 participants differed significantly from the participants that had dropped out of the study: The Wave 10 sample had a significantly higher rate of females than the original sample [*t*(2,470) = 3.435, *p* = 0.001], participants reported significantly lower levels of traditional gender beliefs at age 16/18 [*t*(1,840) = 3.240, *p* = 0.001], and their mothers reported significantly higher educational background [*t*(1,927) = -6.524, *p* = 0.000].

### Measures

All measures were assessed using survey questionnaires. Up to Wave 6 of data collection, participants received and filled out surveys at school. Parents filled out surveys at home. In Wave 10, surveys were mailed to prior participants. In addition, four percent of Wave 10 data were collected through phone interviews and 33 percent of Wave 10 were collected via web search.

#### Traditional Gender Role Beliefs

Participants’ traditional gender role beliefs with regards to job responsibilities were assessed at Wave 5 (age 16) and Wave 6 (age 18) with a 5-item scale (α = 0.83/0.80, e.g., “It is usually better for everyone involved if the man is the achiever outside the home and the woman takes care of the home and family,” see Appendix A). This scale assesses beliefs about the relative importance of a man’s vs. a woman’s career and beliefs about better dispositions of men for career success. The scale was developed by Eccles et al. (1983) and validated in previous studies (Belansky et al., 1993; Frome et al., 1996). Students rated items on a 7-point Likert scale ranging from 1 = Disagree to 7 = Agree. To minimize missing data, missing students’ reports from Wave 6 were supplemented by Wave 5 reports.

#### Participants’ Educational Attainment

At Wave 10 (age 42) participants reported their highest attained educational level (Range: 1 = “12th grade or less” to 10 = “Doctorate degree”). For Wave 10 participants that were located through web search, information was coded using available information.

#### Maternal Educational Attainment

Participants’ mothers were asked to report their highest attained educational level at Wave 1 (Range: 1 = “Grade school” to 9 = “Ph.D or professional degree”). In addition, participants were asked to report their mother’s educational level at Wave 5 (age 16) with responses ranging from 1 = Grade school to 6 = Graduate school. To minimize missing data, parents’ reports from Wave 1 were converted to the 1–6 response scale and supplemented by Wave 5 student reports.

#### Participants’ Occupational STEM Attainment

At Wave 10 (age 42) participants were asked to report their current occupation. If participants were not currently working, they were asked to report their most recent occupation (*n* = 75).

For the present analyses, the open-ended answers were first coded using employment classification standards set by the United States Bureau of Labor Statistics and the 2010 standard occupational classification (SOC) system manual (U.S. Bureau of Labor Statistics, 2010). Next, SOC-coded occupations were further coded for STEM using U.S. Department of Labor’s STEM classification recommendations and subsequently collapsed to three categories to capture the type of STEM-relatedness: traditional STEM-related careers in the physical sciences, engineering, mathematics, computing and technology (PMET; e.g., engineers, surveyors, and mapping scientists, mathematical scientists, physicists, and astronomers, etc); LS (e.g., biology, health sciences, LS; e.g., biologist, physical therapists, nurses, dentists, and veterinarians, etc); and non-STEM. The categorization of non-STEM occupations was guided by our research question and therefore comprised occupations in the social sciences as well all other occupations (including legislators, chief executives and general administrators, teachers, social workers, homemaker, etc). Three dichotomized indicator variables for each of the STEM categories (LS, PMET, and Non-STEM) indicating membership in the respective category (e.g., 1 = LS-related occupation) were computed.

### Statistical Analyses

To investigate the longitudinal associations of traditional gender role beliefs in adolescence with occupational and educational STEM attainment in adulthood for females and males, multi-group manifest path analyses by gender were conducted. In addition, we used the model comparison approach advocated by Judd et al. (1995, 2009) in our analyses, which encourages the use of specific focused comparisons to test specific theoretically derived comparisons. In this case, we conducted three separate path models comparing the different types of STEM-related careers using the following pair comparisons: LS vs. non-STEM, PMET vs. non-STEM, and LS vs. PMET. These comparisons not only allowed us to compare the differentiated STEM careers with non-STEM careers, but also with each other. In the models, educational attainment and STEM occupational attainment in adulthood (Wave 10, age 42) were regressed on traditional gender role beliefs in adolescence (Waves 5 and 6, ages 16 and 18). Educational attainment also predicted STEM occupational attainment. To take into account participants’ educational family background, mother’s educational attainment was included in the model as a covariate of educational attainment and STEM occupational attainment in adulthood. To address the associations with dichotomous STEM categories (LS, PMET and non-STEM), logistic regressions path analyses were estimated using mixture modeling in MPlus 7.1 (Muthén and Muthén, 2013). Separate models for females and males were estimated using the KNOWNCLASS option in MPlus. To address missing data (≤14%), models were estimated using maximum likelihood estimation with robust standard errors as well as Montecarlo integration (Muthén and Muthén, 2013).

To investigate whether gender differences in educational and STEM occupational attainment were mediated by traditional gender role beliefs, separate mediation path analyses were conducted for each of the four outcomes of interest (educational attainment, LS vs. non-STEM, PMET vs. non-STEM and LS vs. PMET). For each set of analyses, gender was used to predict the outcome to test for existing gender differences in a first step. Then, a path model was estimated, in which gender and traditional gender role beliefs predict the outcome. In addition, gender predicted traditional gender role beliefs. In order for mediation to be met, four conditions had to be met: First, gender must be related to the outcome. Second, gender must be related to traditional gender role beliefs. Third, traditional gender role beliefs should be a significant predictor of the outcome. Fourth, gender should no longer significantly predict the outcome. If all four conditions are met, full mediation is supported. If only the first three conditions are met, partial mediation is supported (Hayes, 2009). To test for the significance of the indirect effect of gender on the outcome via traditional gender role beliefs, the MODEL INDIRECT command in Mplus was used. In addition, models were estimated in Mplus using maximum likelihood estimation as well as Montecarlo integration to address missing data and dichotomous outcome variables (Muthén and Muthén, 2013).

## Results

**FIGURE 1:**
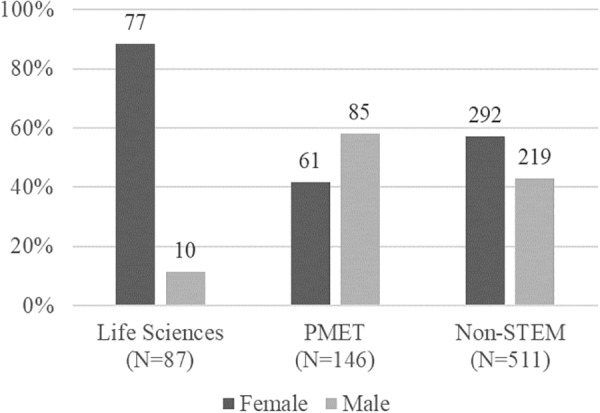
Distribution of STEM-related careers by gender.

Descriptive analyses revealed gender differences in the endorsement of traditional gender role beliefs and educational attainment (see Table 1). Female participants reported lower endorsement of the traditional gender role beliefs scale [*t*(638) = -13.610, *p* = 0.000] in adolescence and higher educational attainment [*t*(689) = 2.964, *p* = 0.003] in adulthood than male participants. The majority of participants were engaged in non-STEM occupations (*n* = 511, 61%) followed by PMET-related (*n* = 147, 18%) and LS-related occupations (*n* = 87, 10%). As shown in Figure 1, gender differences in the distribution emerged. Females were more likely than males to be in LS- related occupations in adulthood [*t*(742) = 6.328, *p* = 0.000], whereas males were more likely than females to be in PMET-related occupations [*t*(742) = -4.422, *p* = 0.000].

**Table 1:** Descriptive statistics for relevant study variables.

	Total (*N* = 744)		Female (*N* = 430)		Male (*N* = 314)		Range
	M	SD	M	SD	M	SD	
*Parent report (Mother)*							
Educational attainment (W1)	3.90	1.12	3.88	1.12	3.92	1.12	1–6
*Participant reports*							
Traditional gender role beliefs (W5/6)	3.09	1.30	2.57	1.03	3.81	1.31	1–7
Educational attainment (W10)	5.90	1.97	6.09	1.93	5.64	1.99	1–10


Table 2 presents correlations of traditional gender role beliefs, educational and occupational attainment, and their mother’s educational attainment separately for males and females. Some gender differences in correlation patterns were evident. For females, traditional gender role beliefs were statistically significantly negatively associated with their mothers’ educational attainment and their own educational attainment as adults. Traditional gender role beliefs among females were also statistically significantly negatively associated with being in a PMET-related occupation, but positively associated with being in a non-STEM occupation. Employment within a LS-related occupation (vs. any other occupation) was not associated with traditional gender role beliefs. Females’ educational attainment in adulthood was positively associated with their mother’s educational attainment and employment in LS- or PMET-related occupations; and negatively associated with being in non-STEM-related occupations. For males, traditional gender role beliefs were also statistically significantly negatively associated with their mother’s educational attainment. Educational attainment in adulthood showed the same correlation pattern as for females: It was positively correlated with mother’s educational attainment and employment in a LS- or PMET-related occupation, but negatively correlated with employment in a non-STEM occupation.

**Table 2:** Bivariate correlations of relevant variables by gender.

	1	2	3	4	5	6
1. Traditional gender role beliefs	–	–0.12	–0.15*	0.02	–0.08	0.07
2. Educational attainment	–0.22***	–	0.26***	0.13*	0.17**	–0.22***
3. Mother’s educational attainment	–0.23***	0.28***	–	0.12	0.02	–0.06
4. LS related occupation (=1)	–0.01	0.13**	0.1	–	–	–
5. PMET related occupation (=1)	–0.13**	0.10*	0.04	–	–	–
6. Non-STEM related occupation (=1)	0.11*	–0.18***	–0.11*	–	–	–


*Coefficients for females below diagonal/coefficients for males above diagonal. ^∗∗∗^*p* < 0.001, ^∗∗^*p* < 0.01, ^∗^*p* < 0.05.*

### Traditional Gender Role Beliefs Predicting Subsequent Educational and STEM Occupational Attainment

**FIGURE 2:**
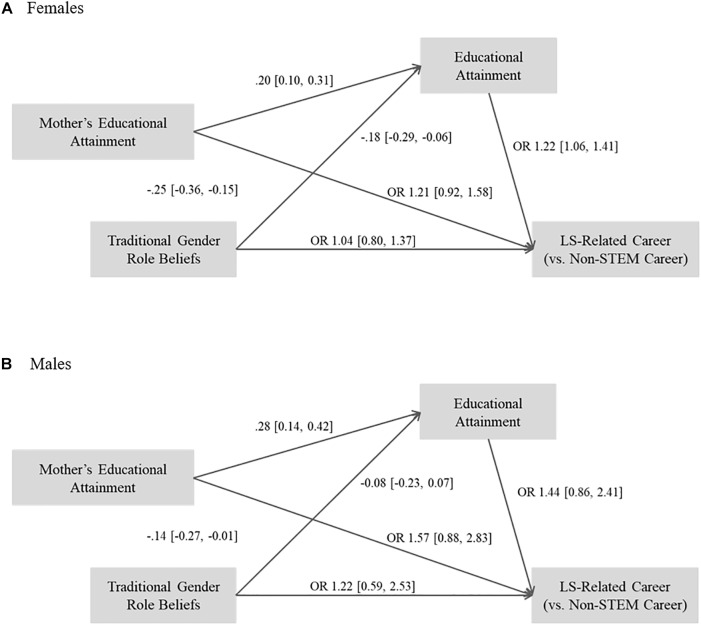
Results of multi-group path analyses by gender for LS-related careers vs. non-STEM careers. **(A)** Results for females and **(B)** results for males. Standardized coefficients are shown for continuous variables. OR, Odds Ratio. 95% Confidence intervals in brackets. Confidence intervals excluding 0/1 for regression coefficients/odds ratios indicate statistical significance.

**FIGURE 3:**
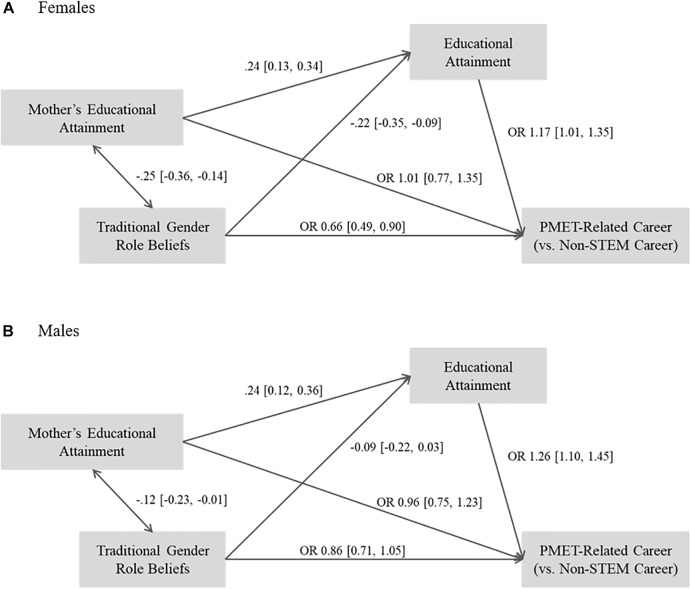
Results of multi-group path analyses by gender for PMET-related careers vs. non-STEM careers. **(A)** Results for females and **(B)** results for males. Standardized coefficients are shown for continuous variables. OR, Odds Ratio. 95% Confidence intervals in brackets. Confidence intervals excluding 0/1 for regression coefficients/odds ratios indicate statistical significance.

**FIGURE 4:**
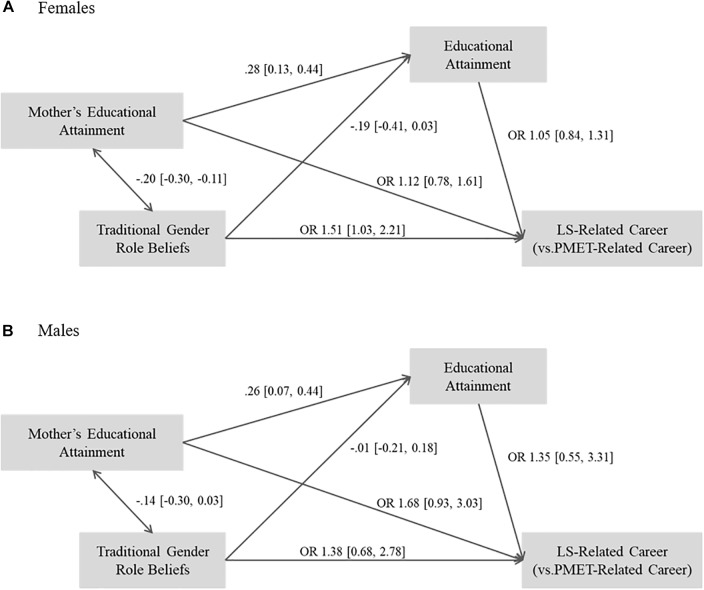
Results of multi-group path analyses by gender for LS-related careers vs. PMET-related careers. **(A)** Results for females and **(B)** results for males. Standardized coefficients are shown for continuous variables. OR, Odds Ratio. 95% Confidence intervals in brackets. Confidence intervals excluding 0/1 for regression coefficients/odds ratios indicate statistical significance.

Figure 2–4 present the results of the multi-group path analyses by gender for each of the STEM category comparisons in our examination of the long-term associations of traditional gender role beliefs with educational and occupational STEM attainment (RQ1).

#### Educational Attainment

With regards to participants’ educational attainment the following pattern was found across all three models (see Figure 2–4): For females, traditional gender role beliefs were significantly negatively associated with mother’s educational attainment and with their own educational attainment in adulthood. In other words, female participants that endorsed stronger traditional gender role beliefs were more likely to have mothers with lower educational attainment and also more likely to attain lower levels of education themselves. Moreover, their educational attainment was statistically significantly and positively associated with their mother’s educational attainment. In other words, females were more likely to attain a higher degree of education when their mothers were also more highly educated. For males, traditional gender role beliefs were marginally negatively associated with mother’s educational attainment. Mother’s educational attainment was also statistically significantly positively associated with males’ own educational attainment in adulthood. However, traditional gender role beliefs were not statistically associated with educational attainment in adulthood for males.

#### STEM-Related Occupational Attainment

With regards to attainment of LS-related occupations in comparison to non-STEM occupations (see Figure 2), traditional gender role beliefs were not associated with attainment of a LS-related occupation for either males or females after taking into account their educational attainment. Educational attainment was statistically significantly associated with a higher likelihood to be in a LS-related career for females, but not males.

With regards to attainment of PMET-related occupations in comparison to non-STEM occupations (see Figure 3), females with more traditional gender role beliefs in adolescence were statistically significantly less likely to be employed in PMET-related careers as adults after controlling for their educational attainment. For males, no statistically significant association of traditional gender role beliefs with the likelihood to be in PMET-related careers was found. Higher educational attainment statistically significantly increased the likelihood for being in a PMET-related career for males and females.

With regards to attainment of LS-related occupations in comparison to PMET-related occupations (see Figure 4), traditional gender role beliefs statistically significantly increased the likelihood of being in a LS-related career instead of a PMET-related career for females. However, higher educational attainment significantly decreased the likelihood of being in a Non-STEM related career for females. The likelihood of being in a LS- vs. a PMET-related occupation was not associated with endorsements of traditional gender role beliefs for males. Moreover, higher educational attainment did not significantly predict the likelihood of being in a LS- vs. a PMET related occupation for either gender.

### Gender Role Beliefs as Mediators of Gender Differences in Educational and STEM Occupational Attainment

To examine whether traditional gender role beliefs explain the gender differences in educational and STEM occupational attainment, separate mediation path analyses were conducted for each of the relevant outcomes (RQ2). Gender was significantly related to all outcomes: Males were more likely to be in a PMET-related career in comparison to a non-STEM career [OR = 1.86, 95% CI (1.28, 2.70)]. In contrast, females were more likely to be in a LS-related career compared to a non-STEM career [OR = 0.17, 95% CI (0.09, 0.34)] as well as when compared to a PMET-related career [OR = 0.09, 95% CI (0.05, 0.20)]. Females also had more years of schooling than males [*b* = -0.11, 95% CI (-0.19, -0.04)].

**FIGURE 5:**
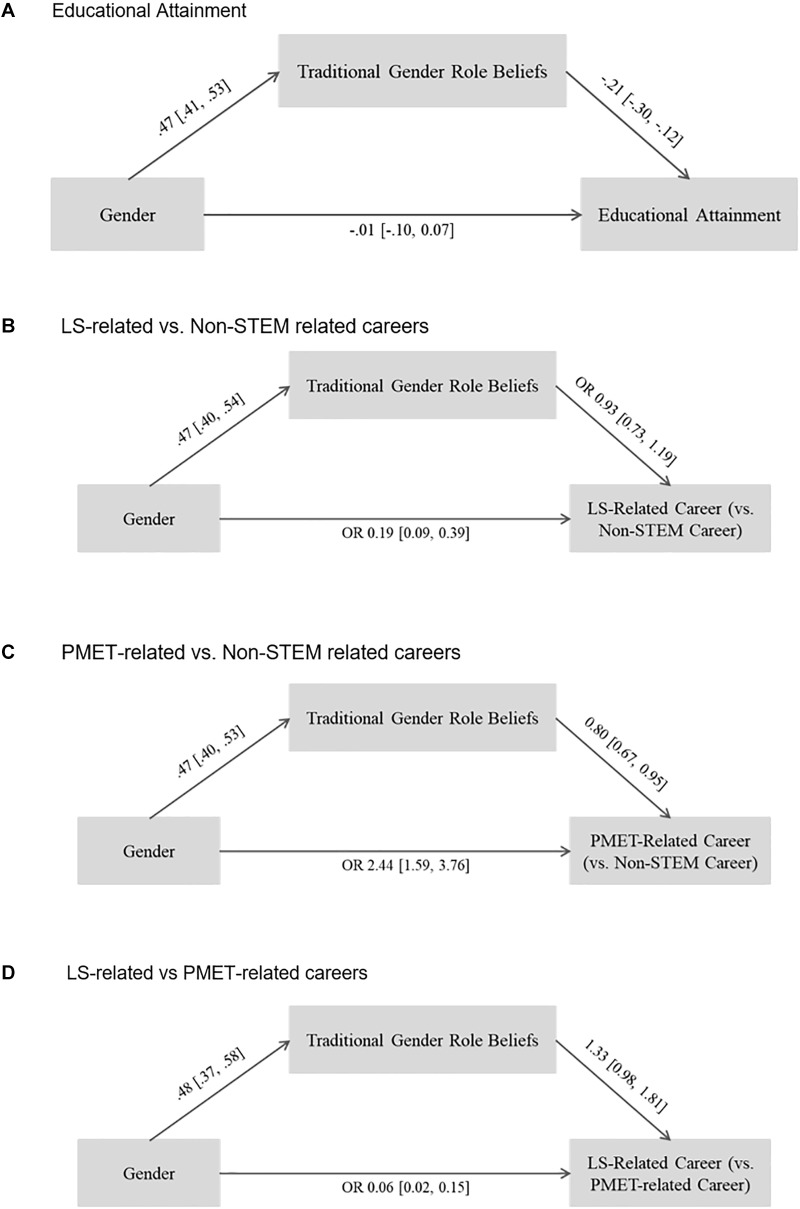
Results of path analyses investigating the mediation of the association of gender with educational and STEM occupational attainment outcomes via traditional gender role beliefs. Gender coded 1, male. **(A)** Results for educational attainment, **(B)** results for comparison of LS-related vs. non-STEM related careers, **(C)** results for comparison fo PMET-related vs. non-STEM related careers, and **(D)** results for comparison of LS-related vs. PMET-related careers. Standardized coefficients are shown for continuous variables. OR, Odds Ratio. 95% Confidence intervals in brackets. Confidence intervals excluding 0/1 for regression coefficients/odds ratios indicate statistical significance.

For educational attainment (see Figure 5A), gender differences in educational attainment were fully mediated by traditional gender role beliefs, as the association of gender and educational attainment was no longer significant after including traditional gender role beliefs as the mediator. In addition, results indicated that the indirect effect was significant [*b* = -0.10, 95% 95% CI (-0.14, -0.05)].

Gender differences in the likelihood to be in a LS-related career vs. a non-STEM career were not statistically significantly mediated by traditional gender role beliefs (see Figure 5B). The association between gender and endorsement of a LS-related career remained significant after including traditional gender role beliefs in the model and no significant association of traditional gender role beliefs with LS-related career attainment was found. Thus, the indirect effect was not significant [OR = 0.92, 95% CI (0.64, 1.20)].

However, gender differences in the likelihood to be in PMET-related career vs. a non-STEM career were partially mediated by traditional gender role beliefs (see Figure 5C). The higher likelihood of males to be in a PMET-related career remained statistically significant after the inclusion of traditional gender role beliefs in the model, but results indicated a statistically significant indirect effect [OR = 0.76; 95% CI (0.61, 0.92)].

Lastly, gender differences in the likelihood to be in a LS- vs. a PMET-related occupation were not mediated by traditional gender role beliefs (see Figure 5D). The higher likelihood of females to be in a LS-related career remained statistically significant after the inclusion of traditional gender role beliefs in the model and no significant association of traditional gender role beliefs and LS-related vs. PMET-related attainment was found. Thus, the indirect effect was not statistically significant [OR = 1.43, 95% CI (0.87, 1.99)].

## Discussion

The current study investigated the impact of traditional work/family related gender role beliefs in adolescence on educational and STEM occupational attainment in adulthood using a longitudinal dataset spanning 20 years. As an important determinant of life choices, traditional work/family related gender role beliefs were used to investigate impacts on educational and occupational attainment in PMET, LS, and non-STEM occupational attainment within and across gender. By doing so, we fill a need for longitudinal studies on the impact of traditional gender role beliefs as well as address the lack of STEM differentiation when investigating its impact on gendered occupational choices in previous research. This is particularly noteworthy given the misrepresentation of women in STEM when LS occupations and PMET occupations are not differentiated. By highlighting these differentiated associations we can better contribute to the conversation of how we can better represent and support females’ STEM-related choices.

### Impacts of Traditional Gender Role Beliefs on Subsequent Educational and STEM Occupational Attainment Within Gender

Our investigation of the impact of traditional work/family related gender role beliefs revealed a nuanced pattern of findings for females. As hypothesized, females with stronger traditional gender role beliefs in adolescence attained lower levels of education in adulthood – a finding that further supports previous work by Scott (2004). One explanation for this association could be that the endorsement of traditional gender roles during adolescence (e.g., beliefs about women’s role as the caretaker at home and in the family) may be a reflection of young women’s expectations for marriage and child bearing early on and their reliance on men’s role to provide financial support as the breadwinner of the family. If so, this explanation would be in congruence with findings by Corrigall and Konrad (2007) that found that women with more traditional attitudes worked fewer hours and had lower income than women with more egalitarian views in their late twenties.

By using a differentiated classification of STEM-related occupations, we also found, as hypothesized, that females’ endorsement of traditional gender role beliefs in adolescence reduced the likelihood of occupational employment within PMET domains, but was not associated with their occupational attainment within LS domains. In addition, more traditional gender role beliefs actually predicted occupational attainment within LS domains over PMET domains. The reduced likelihood of occupational attainment in a PMET domain among females that endorse traditional gender role beliefs lends further support to research that has documented male and female value of gender-stereotyped domains in alignment with their respective gender (e.g., Eccles et al., 1993). However, our nuanced findings with regards to the effects on occupational attainment in PMET- and LS-related careers underline the importance of using a differentiated conceptualization of STEM domains. Endorsement of traditional gender role beliefs did not affect females’ occupational attainment in LS domains negatively. Thus, to truly capture and understand the origins of gender differentiation in the STEM field, a broader conceptualization of STEM-related occupations that is fully inclusive of LS such as health and medicine is needed. This will not only allow for a better scope of STEM-related or, more broadly speaking, science-related occupations, but it will also more accurately represent the participation of women in STEM.

However, it is important to note that our models accounted for females’ educational attainment in adulthood; and for females, their endorsement of traditional gender role beliefs were negatively associated with their educational attainment. It may be that there is an indirect link between traditional gender role beliefs and STEM-typed occupational attainment that is mediated by educational attainment. This might be especially relevant as STEM occupations generally require a higher degree of educational attainment and technical training relative to non-STEM occupations.

Traditional gender role beliefs did not significantly associate with educational or STEM-related occupational attainment for male participants. However, interestingly, associations of traditional gender role beliefs and STEM occupational attainment were in the similar direction as for females, pointing to a similar pattern of impact for males as for females, only less pronounced. Particularly with regards to STEM-related occupational attainment, one reason for the non-significance of the effects for males might be the small sample size of males in LS-related careers. It also needs to be noted that coefficients for females and males were not statistically significantly different from each other.

### Gender Differences in Educational and STEM Occupational Attainment: Impact of Traditional Gender Role Beliefs

Our investigation of whether traditional work/family related gender role beliefs are related to across gender differences revealed that gender differences in the endorsement of traditional gender role beliefs explain differences in the rates of educational attainment and STEM-related occupational attainment of males and females. More specifically, as expected higher educational attainment by females was mediated by lower endorsement of traditional gender role beliefs by females. In addition, as expected stronger endorsement of traditional gender role beliefs by males partially explained a higher rate of attainment of PMET-related careers compared to non-STEM careers. However, gender differences in attainment of LS-related occupations in comparison to non-STEM occupations and PMET-related occupations were not mediated by traditional gender role beliefs. The found effects were, however, in the expected direction and might have been affected by the low sample size of males in LS occupations in the current sample. Thus, in accordance with the Expectancy-Value theoretical framework (Eccles et al., 1983), our study provides some evidence that traditional gender role beliefs are one potential underlying psychological factor that can help explain gender disparity in attainment. This finding further highlights that it is important to have a differentiated conceptualization of STEM occupations, as STEM occupations encompass a variety of occupations with differential values attached to them by males and females.

Given our findings, one potential way to address the existing gender disparity in the traditional STEM fields could be to better contextualize the human applications of these fields to attract more females. It would be equally prudent to address the stereotype of PMET-related occupations as male-typed domains, that are isolating and incompatible with the goals of helping others (Cheryan et al., 2015). This might be deterring females from aspiring to such occupations. On the other hand, our findings indicate that changes in the socialization of societal gendered expectations with a movement to more egalitarian gender role beliefs, as currently ongoing (Brooks and Bolzendahl, 2004), will ultimately help ease gender disparities in educational and STEM occupational attainment.

### Limitations and Future Research

While the longitudinal dataset used in the present study allowed for an investigation of the long-term impact of traditional gender role beliefs, it needs to be kept in mind that the present longitudinal sample was biased toward lower levels of traditional gender role beliefs due to attrition. As a result, the present study did not present the full variation in traditional gender role beliefs that likely exist in the general population. Our present sample was also biased toward having mothers with a higher level of education. Our results, thus, do not represent the full spectrum with regards to participant’s socioeconomic background. Given these constraints with regards to variation in traditional gender role beliefs and socio-economic background, our findings likely underestimate the effects of traditional gender role beliefs on educational and STEM occupational choices. Lastly, the present sample also consisted of a higher rate of females than males due to attrition. As a result, the sample size for individual STEM categories (e.g., LS) was small for male participants. This means that these particular findings need to be interpreted with caution due to the lack of power. To address the bias in our present sample, future research should replicate the findings using a more gender balanced sample capturing effectively the whole spectrum of traditional gender role beliefs, STEM occupations, and socio-economic backgrounds to test generalizability.

Our findings illustrate how general beliefs about societal norms, i.e., traditional gender role beliefs, can affect specific life choices in important life domains, i.e., educational and occupational attainment. Our findings did, however, not look into the educational and occupational trajectories of the participants to see how educational and occupational aspirations and choices developed over time. This important future avenue for research would allow us to better understand the educational and occupational pathways taken by females and males. Such analyses might shine a light on whether females and males differ in the timing or variation of educational and occupational choices, which might, in turn, affect their eventual educational, and occupational attainment.

Future research should also examine the mechanisms through which traditional gender role beliefs affect educational and occupational choices. As previously discussed, traditional gender role beliefs are likely to inform valuing of education and particular STEM domains, which, in turn, determine occupational choices (Wigfield and Eccles, 2000). They might also inform gender-specific stereotypes about women’s lack of competencies in STEM majors and occupations, which have been found to negatively influence STEM choices for women (Nosek and Smyth, 2011; Cundiff et al., 2013). These possible ways through which traditional gender role beliefs might differentially affect educational and occupational choices for females and males, particularly in STEM, need to be empirically tested.

In addition, apart from exploring the processes driving the impact of traditional gender role beliefs on career choices, future analyses should explore how other important life choices (e.g., marriage, children) mediate or moderate the impact of traditional gender role beliefs on educational and occupational attainment. More importantly, particularly life choices with regards to the timing of marriage and child bearing very likely affect educational and occupational pathways differentially for females and males. As such, another significant avenue of research will also be to examine actual, and perceived, opportunities for employment and lifestyle affordances (i.e., number of hours worked, work-life balance) of STEM-related domains by men and women that could contribute to gender-differentiated choices and pathways as a function of their gender role beliefs. For example, women might gravitate more toward LS-typed careers if there are a greater number of opportunities for work in non-academic settings as opposed to traditional science domains (Ceci et al., 2014). Research is beginning to examine the congruence of perceived affordances and desired goals in explaining gender-differentiated STEM occupational choices (e.g., Diekman et al., 2016). It will be imperative to continue this avenue of research and examine how gender roles beliefs inform a socially constructed narrative of perceived abilities, affordances, and anticipated goals and resultant choices, if we are to support continued opting into these STEM fields.

Overall, our findings showcase the importance of culturally socialized general beliefs about society, in this case traditional work/related gender role beliefs, in influencing the specific life choices women and men make, and specifically their potential in explaining disparate gender participation in STEM.

## Ethics Statement

This study was carried out in accordance with the recommendations of the Institutional Review Board of the University of Michigan and the University of California, Irvine, Irvine, CA, United States with written informed consent from all subjects. Written informed consent for subjects under the age of 16 was obtained from parents. All subjects gave written informed consent in accordance with the Declaration of Helsinki. The protocol was approved by the Institutional Review Boards of the University of Michigan and the University of California, Irvine, Irvine, CA, United States.

## Author Contributions

A-LD and NS conceived the idea of the study. JE was the architect of the data used in the study. A-LD conducted the analyses and wrote the manuscript with feedback and assistance from NS and JE.

## Conflict of Interest Statement

The authors declare that the research was conducted in the absence of any commercial or financial relationships that could be construed as a potential conflict of interest.

## Appendix A Traditional Gender Role Belief Scale

In general, men are more reliable on the job than women. In general, men are naturally more competitive than women. It bothers me to see a man being told what to do by a woman. Men are naturally better than women at mechanical things. It is usually better for everyone involved if the man is the achiever outside the home and the woman takes care of the home and family.
